# Effects of Adolescent THC Exposure on the Prefrontal GABAergic System: Implications for Schizophrenia-Related Psychopathology

**DOI:** 10.3389/fpsyt.2018.00281

**Published:** 2018-07-02

**Authors:** Justine Renard, Walter J. Rushlow, Steven R. Laviolette

**Affiliations:** ^1^Department of Anatomy and Cell Biology, University of Western Ontario, London, ON, Canada; ^2^Department of Psychiatry, Schulich School of Medicine & Dentistry, University of Western Ontario, London, ON, Canada

**Keywords:** GABAergic transmission, adolescence, cannabis, prefrontal cortex (PFC), psychiatric diseases

## Abstract

Marijuana is the most commonly used drug of abuse among adolescents. Considerable clinical evidence supports the hypothesis that adolescent neurodevelopmental exposure to high levels of the principal psychoactive component in marijuana, -delta-9-tetrahydrocanabinol (THC), is associated with a high risk of developing psychiatric diseases, such as schizophrenia later in life. This marijuana-associated risk is believed to be related to increasing levels of THC found within commonly used marijuana strains. Adolescence is a highly vulnerable period for the development of the brain, where the inhibitory GABAergic system plays a pivotal role in the maturation of regulatory control mechanisms in the central nervous system (CNS). Specifically, adolescent neurodevelopment represents a critical period wherein regulatory connectivity between higher-order cortical regions and sub-cortical emotional processing circuits such as the mesolimbic dopamine (DA) system is established. Emerging preclinical evidence demonstrates that adolescent exposure to THC selectively targets schizophrenia-related molecular and neuropharmacological signaling pathways in both cortical and sub-cortical regions, including the prefrontal cortex (PFC) and mesolimbic DA pathway, comprising the ventral tegmental area (VTA) and nucleus accumbens (NAc). Prefrontal cortical GABAergic hypofunction is a key feature of schizophrenia-like neuropsychopathology. This GABAergic hypofunction may lead to the loss of control of the PFC to regulate proper sub-cortical DA neurotransmission, thereby leading to schizophrenia-like symptoms. This review summarizes preclinical evidence demonstrating that reduced prefrontal cortical GABAergic neurotransmission has a critical role in the sub-cortical DAergic dysregulation and schizophrenia-like behaviors observed following adolescent THC exposure.

## Introduction

Marijuana is the most widely used illicit drug in the world (1). With more jurisdictions looking to legalize cannabis, there is potential for increasing rates of regular use and dependence within the general population. While cannabis strains popular in the 70's contained much lower levels of its naturally psychoactive compound, Δ9-tetra-hydrocannabinol (THC) (~2–4%), current popular street strains such as “sinsemilla” have been reported to contain THC concentrations of up to 12–18% (2). However, it is still a matter of debate if this increasing content of THC found within popular street strains of cannabis is emerging as a factor in THC-related psychiatric risk; especially if used during adolescence. Indeed, more and more preclinical and clinical studies highlight that adolescent chronic exposure to THC can increase the risk of onset of psychiatric diseases later in life, including schizophrenia (3–7). Chronic marijuana use before the age of 17 and elevated THC concentration in current popular street strains are factors that can increase this risk of developing schizophrenia*. Why is this the case?* Because adolescence is a period of vast neuronal, maturational, and morphological changes throughout the brain. Specifically, the adolescent central nervous system (CNS), in particular the frontal cortex (8, 9), is in a state of extreme vulnerability due to myelination, synaptic pruning, volumetric growth, changes in receptor distribution, and programming of neurotrophic levels (10–13). Nevertheless, given the specific vulnerability of the adolescent brain to THC-induced neuropsychiatric risk, it is critical to identify and characterize the specific mechanisms and neuroanatomical circuits by which exposure to chronic THC may set-up the developing brain for later onset of serious mental health disorders, such as schizophrenia.

The prefrontal cortex (PFC), one of the last areas to reach maturity in the adolescent brain (14), undergoes immense synaptic remodeling and consolidation of neural circuitry between cortical and subcortical structures. This neuronal remodeling of the PFC results in the refinement of the excitatory-inhibitory balance essential for the maturation of normal adult behaviors and cognition (15–17). Importantly, cannabinoid type 1 receptors (CB1Rs), principally localized on PFC GABAergic circuits, are essential for the maturation of the PFC. Specifically, CB1Rs play a crucial role in the maintenance of cortical oscillatory states via homeostatic regulation of the excitatory/inhibitory neuronal activity within the PFC (18–20). Therefore, the action of THC on CB1Rs during neurodevelopment can impair PFC-CB1R signaling and associated GABAergic functionality leading to dysregulation of the normal prefrontal maturation process. These observable deficits in PFC functionality can induce long-term impairments of prefrontal inhibition and synchronized cortical activity states leading to psychopathological disease (16, 21). As a result, the increased susceptibility to affective disorders and mental illnesses, such as schizophrenia, may be due to disturbances in the maturation process of the PFC during adolescence (15).

Importantly, post-mortem analyses of brain tissue extracted from the cortex of patients with schizophrenia showed a decrease in GABA function in the PFC (22). A reduction in mRNA and protein expression of the glutamic acid decarboxylase-67 (GAD67), the enzyme synthesizing GABA neurotransmitter, is observed in the dorsolateral PFC of patients with schizophrenia (23–27) and is associated with a decrease in GABAergic parvalbumin (PV) interneurons. Increased expression levels of prefrontal cortical GABA-A receptor α2 subunits and reduced levels of the α1 and δ subunits (28–30) have also been observed in schizophrenia. This attenuated GABAergic function in the PFC may cause abnormalities in the synchronization of gamma-band, prefrontal neuronal activity, and sub-cortical dopaminergic (DAergic) transmission, which may in turn lead to schizophrenia-like symptoms such as hallucinations as well as pathological affective and cognitive deficits (31, 32).

## Adolescence and maturation of the prefrontal GABA system

The adolescent period is associated with the maturation of cognitive functions, such as working memory, decision-making, and impulsivity control. These cognitive functions are dependent on proper PFC maturation and function, and are essential for the acquisition of adaptive adult behaviors and cognitive processing (11, 15, 33). The human PFC continues to develop throughout adolescence before reaching complete adult maturity at approximately 30 years of age (34). During adolescence, the PFC undergoes massive functional remodeling including refinement of GABAergic functionality and modifications in the excitatory–inhibitory neuronal balance (16, 17). This strong remodeling of brain areas during adolescence is associated with a specific developmental window wherein environmental factors, such as exposure to psychotropic drugs, can profoundly affect the normal trajectories of cortical circuit development, making adolescents highly vulnerable to drug-related developmental disturbances (11, 13, 35).

At the neurochemical level, a variety of neurotransmitter systems undergo major developmental changes in the PFC during adolescence. For example, a massive pruning in the glutamatergic system characterized by a decrease in density of spines on pyramidal cells and synapse elimination of glutamatergic excitatory input is observed in the adolescent PFC (11, 36). In addition, DAergic inputs to the PFC and the activity of the enzyme catechol-O-methyl transferase (COMT) increase during adolescence and decrease thereafter (8, 11, 37, 38). Most importantly, adolescent PFC GABAergic transmission also endures important functional remodeling and plays a key role in the maturation of the PFC (39). Indeed, prefrontal GABAergic maturation during adolescence is a key proponent for consolidation of adult neuronal circuitry in the PFC via a change in the balance of excitatory-inhibitory activity (15, 39, 40). During the transition from adolescence to adulthood in humans, evidence suggests a strong decrease in synaptic activity on GABAergic interneurons within the PFC (11). The most substantial changes in the function of the adult brain involve reorganization of local GABAergic interneurons in the PFC (17, 39). Specifically, the prefrontal GABAergic subpopulation of interneurons expressing calretinin (CR) and parvalbumin (PV) continue to differentiate and mature in the dorsolateral PFC during adolescence (41, 42). Populations of CR- and PV-GABAergic interneurons display opposing developmental patterns characterized by a decrease in CR-GABAergic expression and an increase in PV-GABAergic interneurons expression (17). Given that PV-GABAergic neuronal expression is dependent on glutamatergic transmission (39), it is believed that the increase in PV expression might be associated with the increase in glutamatergic excitatory inputs selectively projecting on fast-spiking PV-GABAergic interneurons during adolescence (17, 39).

At the postsynaptic level, the subunit composition of GABA-A receptors plays a critical role in the adolescent PFC (15, 17, 40). The expression of GABA-A receptors in human subcortical structures reaches levels of maturity at age 14, however, in the frontal cortex and PFC the receptors do not reach adult levels until 18 and 19.5 years of age, respectively (42, 43). Interestingly, distinct developmental patterns of specific GABA-A receptor subunit distribution have been observed at both the mRNA and protein levels in the cortex (44). For example, a shift from GABA-A receptors α2- to α1-subunits, characterized by an increase in α1 subunit expression levels and a concomitant decrease in both α2 and α5 subunits expression levels, occurs in the PFC during adolescence (45, 46). The specific functionality of α1 subunits is to evoke faster decay times and subsequent fast synaptic inhibition (15). Thus, this shift from α2-α1 GABA-A receptor subunits may promote a profound biological effect during adolescent development. Studies in rodents and non-Human primates analyzing the “inhibitory postsynaptic current (IPSC) frequencies onto pyramidal neurons and local field potential recordings of GABA-A-mediated prefrontal responses to afferent drive *in vivo”* confirmed that PFC GABAergic function increases during adolescence (16, 40, 47, 48). According to the authors, this suggests that adolescent PFC inhibition is mostly mediated by fast-spiking GABA interneurons because of their functional prevalence over non-fast spiking GABA interneurons (15, 39).

Human studies show that working memory and executive control, both PFC-dependent cognitive processes, reach adult capacity at the age of ~19 years (49, 50). By controlling the PFC pyramidal neuronal activity, PV-GABAergic interneurons are essential key players in the regulation of working memory, executive functions, and transmission of information between cortical areas (51–53). For example, there is an observed correlation between executive functional performance and maturation of PV-GABAergic neurons in non-human primate models (54–56). Thus, alteration of the GABAergic system during adolescence has the potential to lead to enduring cognitive abnormalities persisting into adulthood. Given the role of PFC GABAergic function during adolescence, any direct or indirect insults that can compromise the role of prefrontal GABAergic interneurons during this critical period might prevent the acquisition of normal PFC inhibitory function and lead to behavioral and physiological abnormalities.

## Schizophrenia and associated GABAergic neuronal alterations

**Figure 1:**
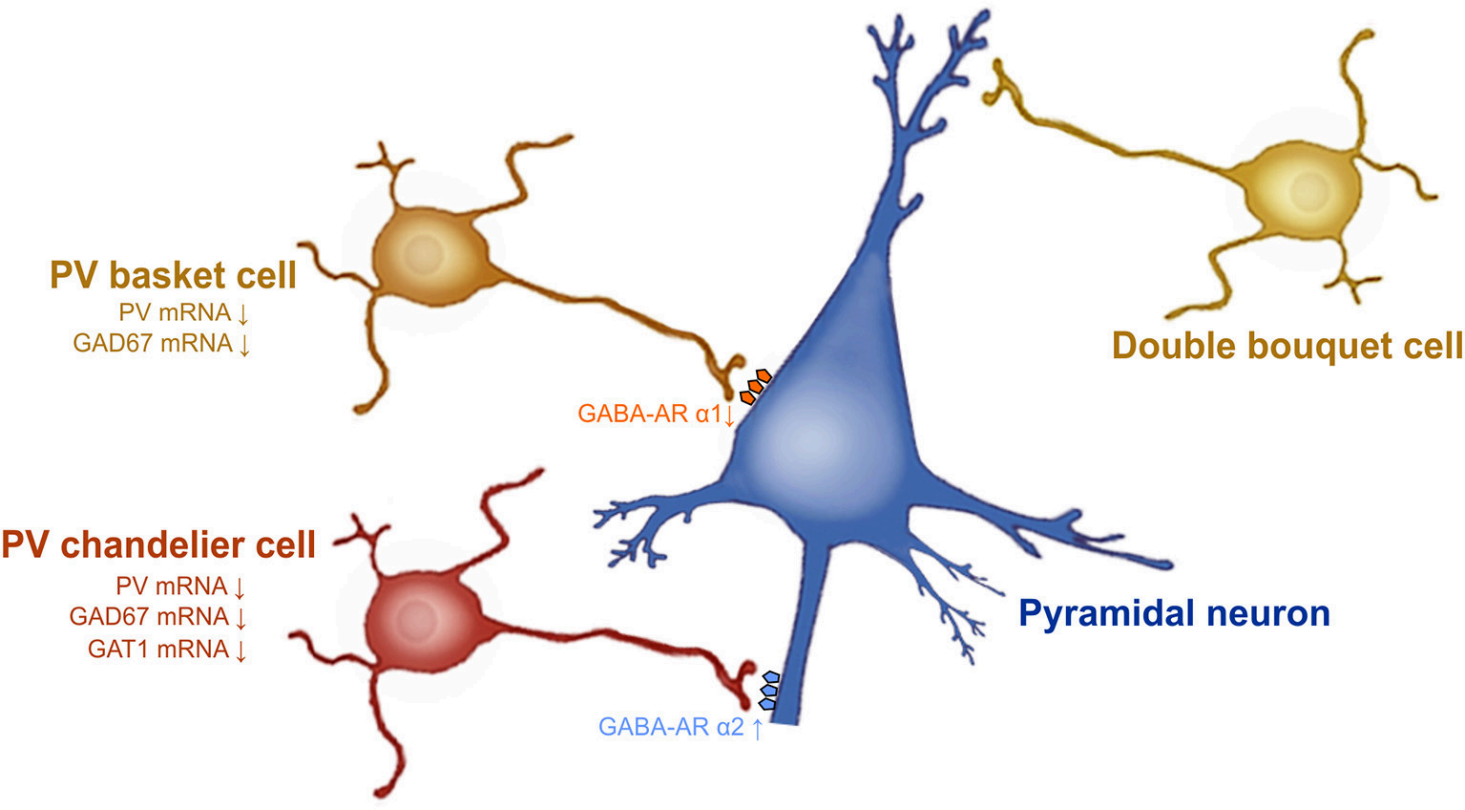
Prefrontal GABAergic function alteration in schizophrenia. GAD67 mRNA expression levels are decreased in PV-GABAergic neurons (i.e., chandelier and basket neurons) in the PFC. GAT-1 mRNA expression levels are reduced in the PV-GABAergic chandelier neurons. GABA-A α2 receptor subunits are increased in the AIS of pyramidal neurons and GABA-A α1 subunits are reduced at synapses from PV-GABAergic basket neurons, as a compensatory response to reduced GABA synthesis and extracellular GABA concentrations. Figure modified from (60).

Schizophrenia is a psychiatric disorder affecting approximately 1% of the population. This devastating psychopathology is characterized by positive symptoms (paranoia, agitation, depersonalization, hallucinations, delusions, dysphoria), negative symptoms (anhedonia, social withdrawal), and cognitive impairments (disorganized thinking, working memory deficits, difficulty concentring, sensorimotor gating deficits). Several lines of evidence demonstrate that dysfunction of PFC GABAergic neurotransmission is a cardinal feature of the pathophysiology of schizophrenia (57). A reduction of GAD 67 mRNA and protein levels is observed in layers 1 through 5 of post-mortem dorsolateral PFC brain tissue from patients with schizophrenia (23, 24, 45, 58, 59). The subpopulation of GABA inhibitory neurons that seems to be particularly involved in schizophrenia is PV-GABAergic neurons (i.e., chandelier and basket neurons) (Figure 1). Indeed, 50% of the PV-GABAergic neurons display strong reduction in GAD67 mRNA expression (24). This reduction in GAD67 mRNA is concomitant with an overall decrease in PV mRNA expression (24, 25). Conversely, the density of PV-and CR-GABAergic neurons (24, 61, 62), as well as CR mRNA expression are unaffected in schizophrenia (24, 63). This suggests that the decrease of PV mRNA expression is not associated with a decrease in the density of PV-GABAergic neurons. The activity of prefrontal pyramidal neurons is strongly inhibited by PV-GABAergic basket and chandelier neurons which establish synapses at the soma and axon initial segment (AIS) of the pyramidal neurons, respectively. As for the double bouquet cells, they mostly establish synapses at the distal dendrites; providing a weaker inhibitory regulation of the pyramidal neurons compared to both basket and chandelier PV-GABAergic neurons (64) (Figure 1). Therefore, a reduction of GAD67 in PV-GABAergic neurons might alter neuronal activity between pyramidal and GABAergic neurons, thus impacting the proper regulatory function of prefrontal excitatory/inhibitory balance (Figure 1).

Besides GAD67, evidence also demonstrates that the GABA transporter GAT-1 plays a role in schizophrenia. For example, GAT-1 mRNA and protein expression are reduced in GABAergic neurons in the dorsolateral PFC of patients with schizophrenia (65, 66). Considerable evidence indicates that reduced GAT-1 levels associated with schizophrenia occur in PV-GABAergic chandelier neurons (57, 60, 66) (Figure 1). First, reduction of GAT-1 mRNA expression levels are found in the same cortical layers where the density of PV-GABAergic neurons is found to be decreased (i.e., layers 2 through 5) (66, 67). In addition, patients with schizophrenia display reduced density of GAT-1 containing PV-chandelier GABAergic neurons within the dorsolateral PFC. The strategic presynaptic localization of GAT-1 confers the role of GABA reuptake in the synapse (66, 68) and regulates the duration and efficacy of extracellular GABA levels (69). In addition, GAT-1 actively participates in GABA-A receptor-mediated phasic and tonic inhibition on neuronal activity (69). Therefore, changes in GAT-1 function in the PFC can undoubtedly lead to alteration of synaptic GABAergic neurotransmission; contributing to schizophrenia psychopathology.

The comprehension of the postsynaptic GABA-A receptor expression levels will help with understanding the consequences of these presynaptic cortical alterations. At the postsynaptic level, most of the physiological actions of GABA are induced by the GABA-A receptors (70). GABA-A receptors are ligand-gated chloride ion channels. They are pentameric receptors composed of α1–6, β1–3, γ1–3, δ, ε, π, and θ (71) subunits. The most common pentameric subunits are comprised of 2α, 2β, and 1γ or 1δ subunits (72). In the GABAergic synapses, GABA-A α2 subunits are mostly localized on the AIS of pyramidal neurons (60, 73). GABA-A α2 subunits which possess a high affinity for GABA neurotransmitter, are characterized by fast activation and slow deactivation (74). Thus, the functions and specific localization of GABA-A α2 subunits on the AIS of pyramidal neurons have a crucial role for inhibiting pyramidal neuron activity (75). An upregulation of the GABA-A α2 receptor subunit has been found in the AIS of pyramidal neurons in schizophrenia (72) (Figure 1). Interestingly, in schizophrenic individuals the increased expression of GABA-A α2 receptor subunits is correlated with GAT-1 reductions (76) and seems to be unrelated to recent use of antipsychotic medications (77). PV-GABAergic neurons, which project on the AIS of pyramidal neurons, display decreased expression levels of GAD67 and GAT-1 mRNAs in schizophrenia (60). Reductions in GAD67 lead to reduced GABA synthesis and GABA release (78, 79). Therefore, it is believed that this increase in GABA-A α2 subunit density at the AIS of pyramidal neurons and the reductions in GAT1, and PV at the presynaptic level, may be a compensatory mechanism in response to reduced GABA synthesis and GABA release from PV-GABAergic chandelier cells (Figure 1). Importantly, these changes in GABA-A α2 expression are not found in subjects suffering from other psychiatric disorders and seem to be specific to schizophrenia psychopathology (77). Furthermore, post-mortem studies of recently deceased patients with schizophrenia showed that GABA-A α1 and δ subunits were reduced in the dorsolateral PFC. These reductions were not associated with a recent use of antipsychotic medication (80). Given that GABA-A α1 subunit receptors are predominant at synapses from mature PV-GABAergic basket cells (72), this reduction of GABA-A α1 subunits observed in schizophrenia confirm the weaker GABAergic synaptic transmission from those cells (72). Importantly, the reduced levels of GAD67, PV, GAT-1, and the GABA-A receptor subunits α1 and δ levels in schizophrenia are found, not only in dorsolateral PFC, but also in other brain regions such as the primary visual cortex, anterior cingulate, and primary motor cortices (72, 81). This indicates that GABA neurotransmission dysfunction may underlie the diverse range of symptoms of schizophrenia that comprise a cluster of perceptual, motor, and cognitive symptoms (72, 82).

## Consequences of altered gabaergic function in pv-neurons in the dorsolateral PFC in schizophrenia

It is well established that working memory impairments are core cognitive deficits in schizophrenia (83). Working memory performances are associated with proper prefrontal GABAergic signaling from PV-GABAergic neurons projecting on the perisomatic region of pyramidal neurons (79). Therefore, reduced prefrontal PV-GABAergic neuronal activity can lead to working memory deficits, a core symptom of schizophrenia pathology (84). In addition, the interaction between PV-GABAergic neurons (both chandelier and basket neurons) and pyramidal neurons in healthy brains is essential for the induction of synchronized gamma oscillations, at frequencies comprised between 30 and 80 HZ (83, 85–87). Neural oscillations and synchronization of cortical networks permit proper communication between cortical structures and represents an essential mechanism to support synaptic plasticity and diverse higher-order cognitive functions, such as learning and memory, executive functions, attention and, consciousness (88). Increasing evidence shows that altered PFC gamma oscillations is a cardinal pathological feature of schizophrenia. For example, impaired PFC gamma oscillations are observed in the first psychotic episode of schizophrenia (89). In addition, schizophrenic patients present abnormal synchronization between cortical areas and attenuated gamma band oscillations (frequency range between 30 and 200 Hz) during visual perception and during higher order cognitive tasks analyzing working memory and executive functions (55, 88, 90–93). The reduced gamma oscillatory activity was correlated with reduced performances in the different cognitive tasks. Patients with schizophrenia also present attenuated oscillations in the beta and theta frequency ranges during the 3 phases of working memory processes (i.e., encoding, maintenance, and retrieval) (72, 82). Conversely, other studies have reported that patients with schizophrenia display enhanced gamma oscillatory activity during working memory tasks (94, 95), which may be associated with reduced GABAergic interneuron transmission in cortical circuits and altered function of PV-positive interneurons (96–98).

It is believed that abnormal gamma band oscillations are associated with the severity and nature of the different symptoms of schizophrenia. Indeed, enhanced cortical gamma band oscillatory activity in schizophrenia may be correlated with increased positive symptoms such as hallucinations and reality distortion (99, 100). Conversely, attenuated cortical gamma band oscillatory activity in schizophrenia may be correlated with negative symptoms of schizophrenia such as affective, emotional, and social cognition disturbances (101, 102). Overall, considerable evidence demonstrates that dysregulated gamma band oscillatory activities, resulting from impaired regulation of pyramidal cell firing synchronization from PV-GABAergic inhibitory neurons, may lead to diverse symptoms of schizophrenia, including episodic and working memories deficits, as well as affective and social behavior impairments.

## Adolescence cannabis use, GABAergic system and schizophrenia

The use of high-THC marijuana strains is correlated with elevated rates of transient psychotic episodes and poorer clinical outcomes in psychiatric patient populations (103). A case-control study showed that daily use of high-THC marijuana strains increases the risk of developing a psychotic disorder by five compared to non-users of cannabis (104). Clinical studies have showed that THC and other cannabinoids can induce transient positive and negative symptoms and mimic some of the neurophysiological and cognitive schizophrenia-like abnormalities. For example, intravenous administration of THC to healthy adult subjects caused schizophrenia-like positive and negative symptoms (105). The perceived THC-induced positive symptoms included euphoria, paranoia, feelings of unreality depersonalization, thought disturbances, conceptual disorganization, illusion, and sensorimotor gating deficits. The perceived THC-induced negative symptoms included attenuation of emotional responses, emotional withdrawal, and lack of motivation. In addition, administration of THC to healthy adult individuals induced various cognitive deficits in working memory, verbal fluency, memory recall, attention, and inhibitory control (105, 106). Finally, administration of THC to patients with schizophrenia intensifies their positive and cognitive symptoms (107). On the other hand, it is important to note that the major non-psychoactive component of cannabis, cannabidiol (CBD), can induce opposite effects to THC and has demonstrated antipsychotic and antiepileptic properties. Indeed, recent clinical and preclinical evidence have demonstrated that CBD is able to prevent THC-induced psychotic-like effects and improve positive symptoms of schizophrenia without inducing the well- known deleterious side effects provoked by classical antipsychotics (108–116). CBD has also been shown to be an effective treatment for epilepsy, with very few adverse effects (117). However, the phytochemical profile of illicit cannabis has profoundly changed over the past three decades: with THC content dramatically increasing along with a relative decrease in CBD content (2).

Importantly, from a neurodevelopmental perspective, increased exposure to high-THC cannabis strains among teens is of crucial significance given that several large epidemiological studies have reported increased risk of developing severe cognitive deficits and psychiatric disorders in adulthood, including schizophrenia (7). This risk appears to be higher if the use of cannabis occurs before age of 17. *What neuropharmacological processes underlie these effects?* THC, the primary psychoactive component of cannabis acts on CB1Rs, which play a crucial neurodevelopmental role in the maturation of the adolescent CNS and contribute to the emergence of adaptive adult cognition and affective regulation (19, 20). It is therefore possible that THC-induced dysregulation of CB1R signaling during adolescence may impact CB1R-mediated neural maturational processes, leading to persistent deleterious consequences on adult brain function. As discussed previously, adolescent brain development in humans and animals involves massive synaptic remodeling and pruning (10, 11, 13). This critical period also involves the formation of new neural pathways between cortical regions and sub-cortical areas, such as the limbic/mesolimbic systems which are responsible for emotional, cognitive, and affective processing and wherein CB1Rs are found in abundance (19, 20, 118). Therefore, THC-induced disruption of this cortical-subcortical neurodevelopmental interplay may underlie long-term neuropsychiatric disturbances in adulthood.

**Figure 2:**
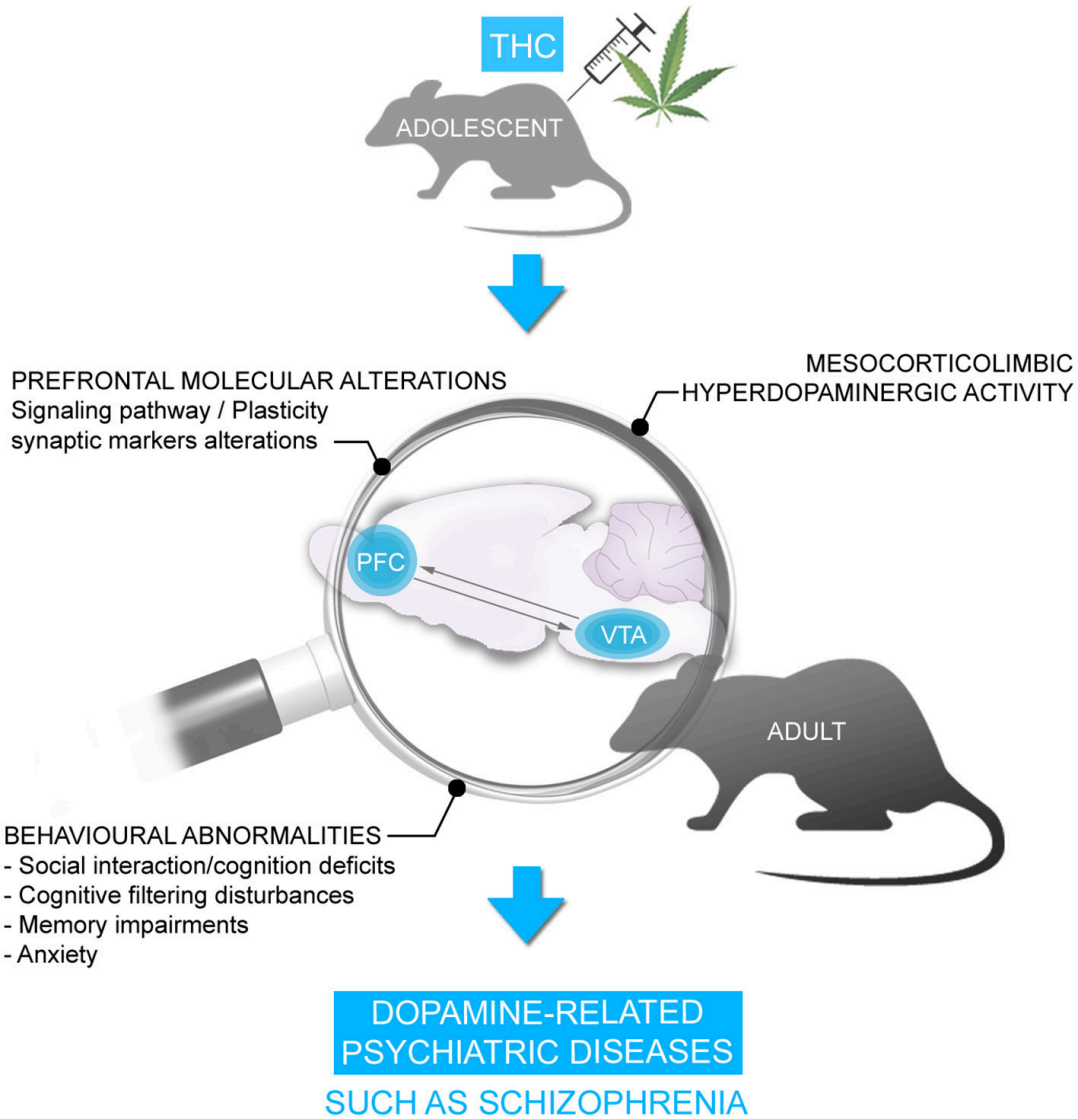
Chronic THC exposure during adolescence is associated with persistent behavioral disorders in adulthood including social interaction/cognition deficits, cognitive filtering disturbances, memory deficits and anxiety. These behavioral disorders were accompanied by alterations in signaling pathway and synaptic plasticity markers and hyperDAergic activity in the mesocorticolimbic pathway, resembling schizophrenia.

Consistent with this clinical data, we and others have previously demonstrated in rodent neurodevelopmental models that adolescent exposure to THC or other CB1R agonists produce several behavioral abnormalities persisting into adulthood, similar to symptoms observed in psychiatric diseases, notably schizophrenia. These behavioral abnormalities included significant social interaction and social cognition deficits, memory impairments, cognitive filtering deficits, and increased anxiety (119–129) (Figure 2). Interestingly, the administration of a mixture of both CBD and THC in adolescent male mice was able to prevent the persistent behavioral abnormalities induced by THC (130). Importantly, many of the extant preclinical studies have been conducted exclusively on male rodents. Given that the deleterious effects of THC can vary depending on sex, particularly in measures of emotional and cognitive function (131, 132), future studies are required to better understand the possible mechanistic role(s) of sex differences in these effects.

We have demonstrated previously that the behavioral deficits induced by THC are associated with a profound hyper-DAergic neuronal state in the ventral tegmental area (VTA), characterized by hyperactive frequency and bursting levels in A10 DA neurons, consistent with mesolimbic dysregulation found in schizophrenia. In addition, we have observed long-term molecular alterations in several schizophrenia-related PFC signaling pathways, including profound reductions in the glycogen-synthase-kinase-3 (GSK-3), p70S6-kinase, Akt (protein kinase B), and mammalian target of rapamycin (mTOR) molecular phosphorylation cascades (122, 123), which have been consistently associated with dysregulation of DAergic function and neuropsychiatric disorders (108, 116, 133–136) (Figure 2). However, *what specific neurodevelopmental mechanisms may underlie the development of these abnormal behavioral, molecular, and neuronal phenotypes following exposure to chronic THC?*

Considerable clinical and pre-clinical evidence points to cannabinoid-mediated modulation of GABAergic function as a critical feature underlying the neuropsychiatric side-effects of THC. For example, CB1Rs are found in abundance on the axon terminals of cortical *GABA* basket neurons, which selectively target cell bodies of pyramidal neurons (84). Under basal conditions, activation of CB1Rs by exogenous cannabinoids suppresses GABA release (137), consequently decreasing inhibition of the PFC. Thus, chronic exposure to cannabis can induce profound reductions in GABA synthesis in cortical GABA basket neurons, which can ultimately lead to increasing the risk and severity of schizophrenia. Moreover, the strategic location of CB1Rs on PFC GABAergic neurons confers a crucial role in the maintenance and control of neuronal network oscillations and homeostatic regulation of the excitatory/inhibitory neuronal activity of CB1Rs within the PFC (18–20). Indeed, cannabis users show decreased theta oscillations that are correlated with working memory deficits (138). In addition, dose-dependent use of cannabis attenuates event-related potential amplitudes in an auditory task assessing attention and cognition, suggesting a decrease in network responses to a stimulus (139).

**Figure 3:**
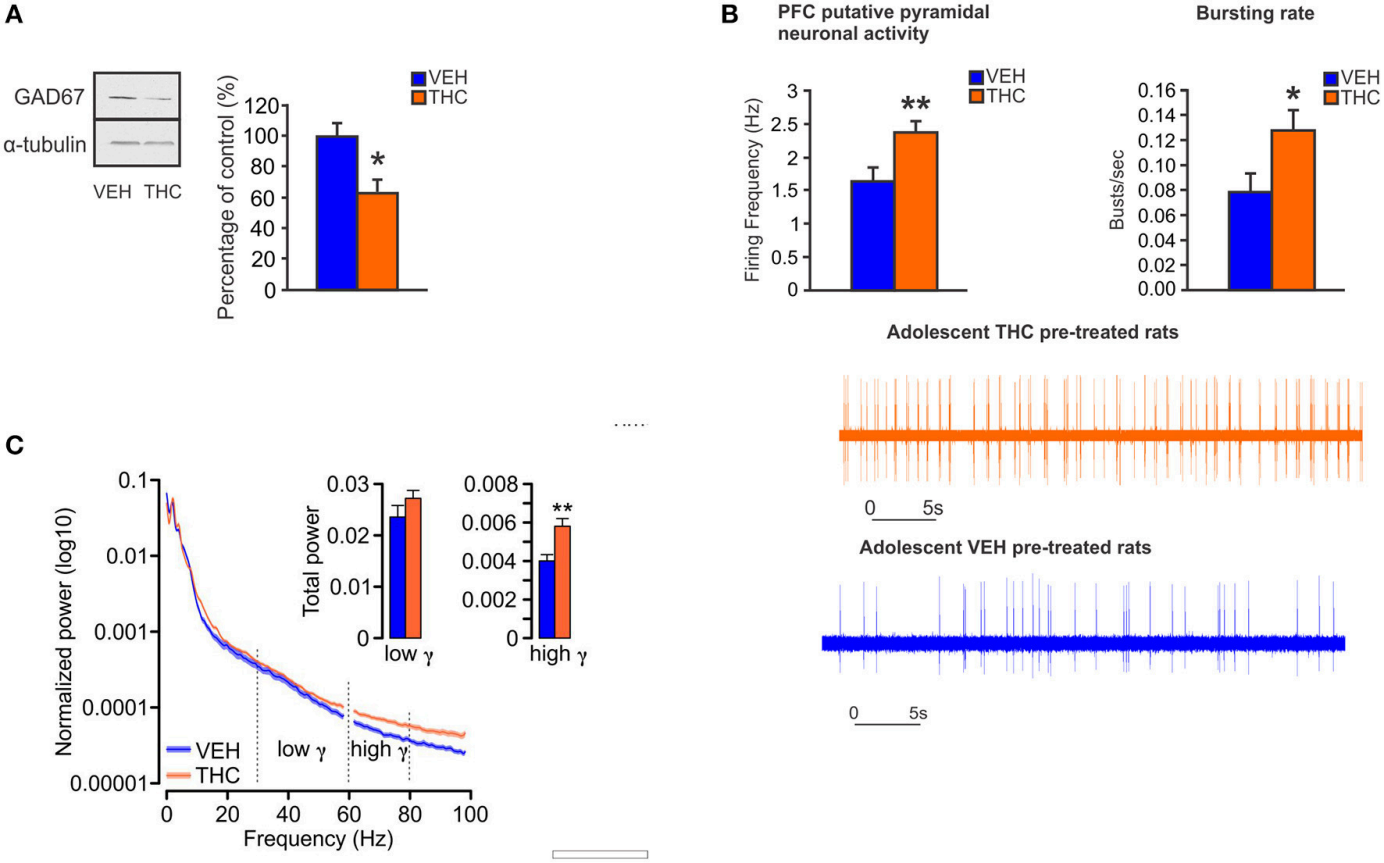
Chronic THC exposure during adolescence induces GABA hypofunction in the adult PFC. **(A)** In adolescent THC pretreated animals, expression level of GAD67 is decreased in the adult PFC as compared to adolescent VEH pretreated animals. **(B)** In adolescent THC pretreated animals, pyramidal neuronal activity is increased in the adult PFC as compared to adolescent VEH pretreated animals. **(C)** In adolescent THC pretreated animals, high gamma (61–80 Hz) bands are increased in the adult PFC as compared to adolescent VEH pretreated animals. Figure modified from Renard et al. (148). *P < 0.05; **P < 0.01.

In animal models, CB1R activation by acute or chronic cannabinoids in the PFC induces alterations of cortical oscillations in theta and gamma bands (140–142). Moreover, systemic administration of CB1R agonists reduces prefrontal and hippocampal gamma and theta oscillations, an effect that is concomitant with spatial working memory impairments (143). In anesthetized rats, activation of CB1Rs by THC within the PFC, not only reduces GABA release but also increases DA and glutamate levels (144). Therefore, similar to schizophrenia-related neuropathology, CB1R activation by exogenous cannabinoids can disrupt cortical network dynamics, and consequently the function of neuronal circuits involved in higher cognitive functions. Furthermore, reduced GABAergic inhibitory activity on pyramidal neurons in the PFC can lead to dysregulated prefrontal pyramidal neuronal networks and impaired gamma oscillatory activities. This may provoke sub-cortical dysregulation of DAergic transmission, and associated schizophrenia-like affective and cognitive deficits (32, 145–147), such that a loss in intrinsic PFC inhibitory substrates may lead to hyper-drive of PFC > VTA outputs, resulting in sub-cortical hyperactivity of mesolimbic DA states. Indeed, given the bi-directional efferent and afferent relationships between the PFC and VTA, our previously described molecular adaptations in the PFC are consistent with subcortical hyperDAergic drive to the PFC. For example, increased activation of DA D2Rs in the PFC is associated with profound downregulation of GSK-3 phosphorylation states (111), consistent with our observed PFC phenotypes following adolescent THC exposure. In addition, we recently reported that chronic THC treatment in adolescent rats induced long-term neuronal and behavioral abnormalities into adulthood that were associated with hypofunction of GABAergic neurotransmission in the adult PFC (148). Specifically, at the behavioral level, adolescent THC pre-treated rats displayed memory impairments, deficits in social interaction and social cognition, anxiety, and amotivational behavior (148). At the neuronal level, adolescent THC treatment induced persistent hyperDAergic activity in the adult VTA, concomitant with increased bursting and firing neuronal activity of pyramidal cells and enhanced prefrontal gamma oscillatory activities in the adult PFC (148) (Figure 3). Chronic THC treatment during adolescence also induced a profound decrease of GAD67 in the adult PFC (148) (Figure 3), consistent with the previous findings from Zamberletti *et al*. showing that adolescent THC chronic exposure reduced prefrontal basal GABA levels and GAD67 expression in PV and cholecystokinin (CCK) GABAergic neurons in adulthood (129).

**Figure 4:**
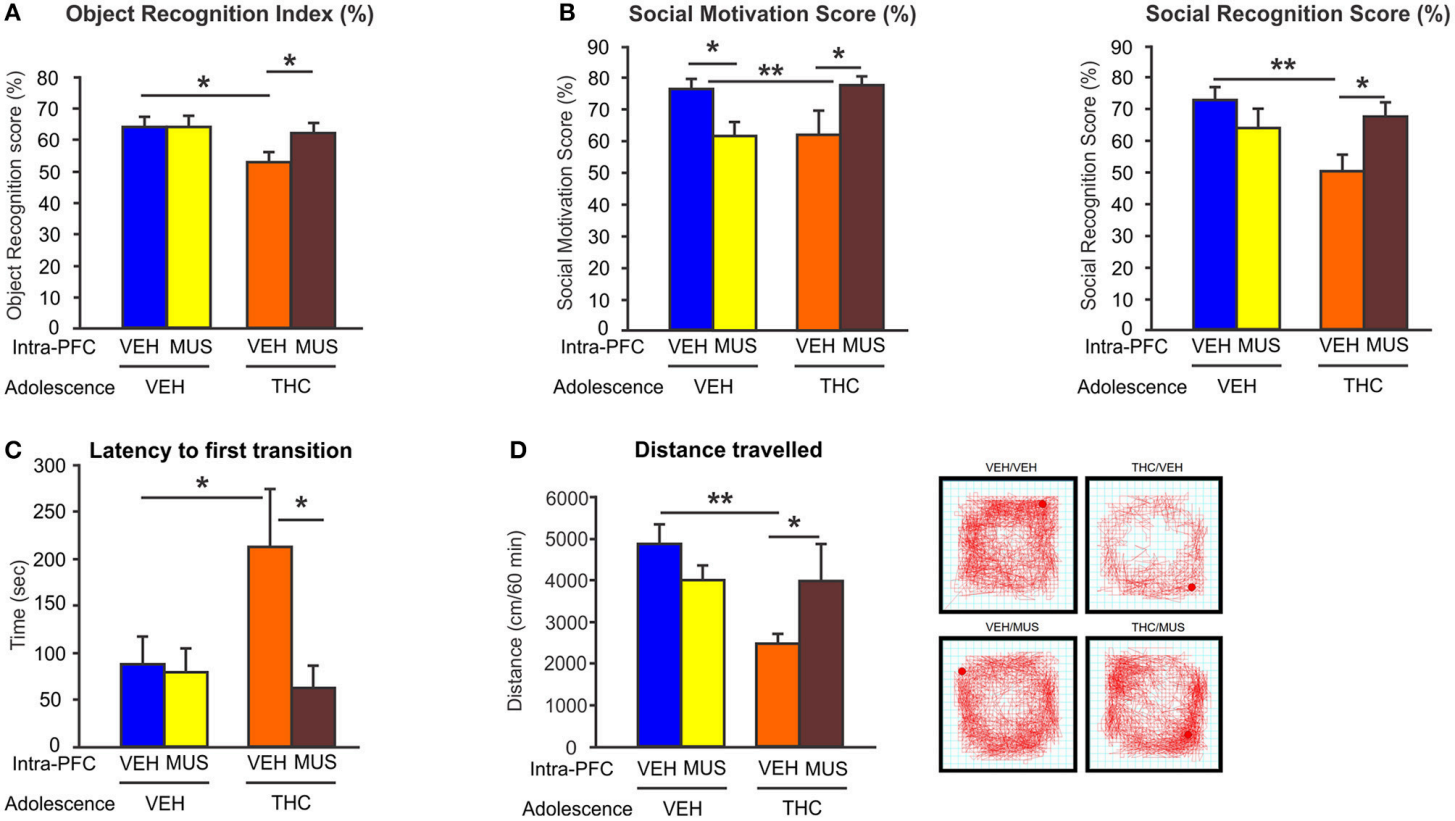
Effects of microinfusion of muscimol (MUS) within the PFC on adolescent THC-induced behavioral abnormalities. **(A)** Adolescent THC pretreated rats displayed short-term memory deficits. Microinfusions of MUS within the PFC restored short-term memory deficits as compared to intra-PFC VEH controls in the object recognition task during adulthood. **(B)** Adolescent THC pretreated rats displayed deficits in social motivation (left) and social cognition (right). Microinfusions of MUS within the PFC improved social motivation (left) and social cognition (right) as compared to intra-PFC VEH controls in the social interaction task during adulthood. **(C)** Adolescent THC pretreated rats displayed increased anxiety. Microinfusions of MUS within the PFC decreased anxiety levels as compared to intra-PFC VEH controls in the light dark box task during adulthood. **(D)** Adolescent THC pretreated rats displayed hypolocomotor activity. Microinfusions of MUS within the PFC normalize locomotor activity as compared to intra-PFC VEH controls in the open field task during adulthood. Figure modified from Renard et al. (148). *P < 0.05; **P < 0.01.

Importantly, we demonstrated that activation of GABA-A receptor with muscimol in the PFC, was able to restore the behavioral and DAergic neuronal abnormalities induced by adolescent THC exposure (148) (Figure 4). These novel data identified a direct mechanism between prefrontal GABAergic hypofunction and dysregulation of DAergic neurotransmission in sub-cortical areas; both phenomena being cardinal features of schizophrenia psychopathology. Consistent with our findings, another study has demonstrated that the COMT genotype, in association with adolescent THC exposure, can modulate both cortical GABAergic and mesocortical DAergic neuronal structure and function. Specifically, COMT KO mice that have been exposed to THC during adolescence, showed more schizophrenia-like neuronal changes as compared to their WT counterparts, including excessive DAergic activity in the mesolimbic system as well as increased density of cortical GABAergic neurons (149). This indicates and confirms that there are functional interactions between GABAergic and DAergic systems within the mesocorticolimbic pathway following adolescent THC exposure. Finally, our recent findings demonstrated that prefrontal GABAergic hypofunction can induce persistent disinhibition of the PFC and lead to pathological abnormalities consistent with PFC dysregulation, similar to those observed in psychiatric disorders, such as schizophrenia (148). Similarly, a recent interesting study showed that adolescent THC exposure in female rats caused reduced expression of several genes involved in synaptic plasticity within the PFC, including markers for the glutamatergic and GABAergic systems (i.e., *Grm3, Gabra, Abat*, and *Dlg4* genes) (150). Interestingly, these genes have been implicated in schizophrenia and mood disorders (150).

Together, these findings underscore the important functional role of persistent excitatory/inhibitory neuronal activity imbalance within the PFC following adolescent THC exposure. These PFC-related adaptations following THC exposure likely serve as critically important mechanisms underlying the affective and cognitive abnormalities following adolescent THC exposure.

## Conclusions and future directions

Adolescence represents a critical period of neurodevelopment during which external stimuli can persistently change brain function. Overstimulation of CB1Rs by THC during this sensitive developmental period could interfere with normal CB1R-mediated developmental processes and the maturation of PFC GABAergic neurons, thereby leading to long-term dysfunction in prefrontal excitatory/inhibitory (Glutamate/GABA) balance, desynchronization of PFC neuronal networks, and deficits associated with schizoaffective disorders. Prefrontal hypofunction of GABAergic signaling is a cardinal pathological feature of schizophrenia and seems to be a mechanism underlying the neuronal and behavioral disturbances induced by chronic THC exposure during adolescence. Thus, there are profound clinical and public health policy implications from these studies in terms of limiting adolescents to cannabis exposure and/or synthetic compounds that act as direct agonists at the CB1R. In particular, cannabis strains (e.g., sinsemilla) or consummation formats (e.g., dabs or shatter) that may contain particularly high concentrations of THC may be particularly neurotoxic during adolescent neurodevelopmental windows.

Although most preclinical studies involving adolescent THC exposure have highlighted the enduring long-lasting effects of neurodevelopmental THC exposure into adulthood, our most recent evidence suggests that the detrimental persistent effects of THC during adolescence on the adult mesocorticolimbic pathway may be reversed by restoration of prefrontal GABAergic neurotransmission in adulthood. Therapeutic benefits of GABAergic compounds have been demonstrated in clinical studies by their abilities to improve hallucinations, hyperactivity, cognitive deficits, and anxiety in patients with psychiatric disorders, including schizophrenia (151, 152). For example, chronic treatment with a GABA-A α2/3-selective agonist in patients with schizophrenia, attenuates attentional and working memory deficits and normalize the associated abnormalities in gamma oscillatory activities (153). In translational animal models of schizophrenia, activation of GABA-A receptors with the partial agonist imidazenil, is able to restore deficits in social interaction and prepulse inhibition induced by methionine in mice (154). In primates, acute GABA-A receptor activation with a GABA-A α2/3-selective agonist blocked ketamine-induced cognitive deficits (72). Moreover, absence of α2/3 subunits in mice caused a hyperDAergic state concomitant with deficits in prepulse inhibition which could be reversed with haloperidol treatment (155). Together, the body of complimentary clinical and preclinical evidence highlighted above, underscores the critical importance of continuing research to characterize the precise neuronal, molecular, and behavioral mechanisms associated with neurodevelopmental THC exposure and associated mental health risks. In addition, the identification of these mechanisms provides the potential for the development of safer cannabinoid-derived formulations using THC and/or the development of novel THC formulations containing neuroprotectants to mitigate these neuropsychiatric risks.

## Author contributions

The manuscript was written by JR. SL and WR supervised the manuscript.

### Conflict of interest statement

The authors declare that the research was conducted in the absence of any commercial or financial relationships that could be construed as a potential conflict of interest. The reviewer GT and handling Editor declared their shared affiliation.
